# Wearable Electrospun Piezoelectric Mats Based on a PVDF Nanofiber–ZnO@ZnS Core–Shell Nanoparticles Composite for Power Generation

**DOI:** 10.3390/nano13212833

**Published:** 2023-10-26

**Authors:** Nehal Ali, El-Refaie Kenawy, A. A. Wadoud, M. I. Elhadary

**Affiliations:** 1 Department of Engineering Physics and Mathematics, Faculty of Engineering, Tanta University, Tanta 31527, Egypt; 2 Polymer Research Group, Department of Chemistry, Faculty of Science, Tanta University, Tanta 31527, Egypt; 3 Atomic Reactors Department, Egyptian Atomic Energy Authority, Inshas, Cairo 13759, Egypt; 4 Department of Mechanical Power Engineering, Faculty of Engineering, Tanta University, Tanta 31527, Egypt

**Keywords:** PVDF, ZnO@ZnS core–shell nanoparticles, electrospinning, piezoelectric

## Abstract

This work adopted a strategy to use new functional high-performance piezoelectric materials for sustainable energy production in wearable self-powered electrical devices. An innovative modification in electrospinning was used to produce highly aligned nanofibers. In the nanogenerator, the flexible membrane constituents were tunefully combined. The novel composite nanofibers were made of Poly (vinylidene fluoride) PVDF, loaded with ZnO@ZnS core–shell nanoparticles to achieve a non-brittle performance of the hetero nanoparticles and piezoelectric polymer. A nanofiber mat was inserted between two thermoplastic sheets with conductive electrodes for application in wearable electronic devices. Complete spectroscopic analyses were performed to characterize the nanofiber’s material composition. It is shown that the addition of 10 wt % ZnO@ZnS core–shell nanoparticles significantly improved the piezoelectric properties of the nanofibers and simultaneously kept them flexible due to the exceedingly resilient nature of the composite. The superior performance of the piezoelectric parameter of the nanofibrous mats was due to the crystallinity (polar β phase) and surface topography of the mat. The conversion sensitivity of the PVDF device recorded almost 0.091 V/N·mm^3^, while that of the PVDF—10 wt % ZnO@ZnS composite mat recorded a sensitivity of 0.153 V/N·mm^3^, which is higher than many flexible nano-generators. These nanogenerators provide a simple, efficient, and cost-effective solution to microelectronic wearable devices.

## 1. Introduction

**Figure 1:**
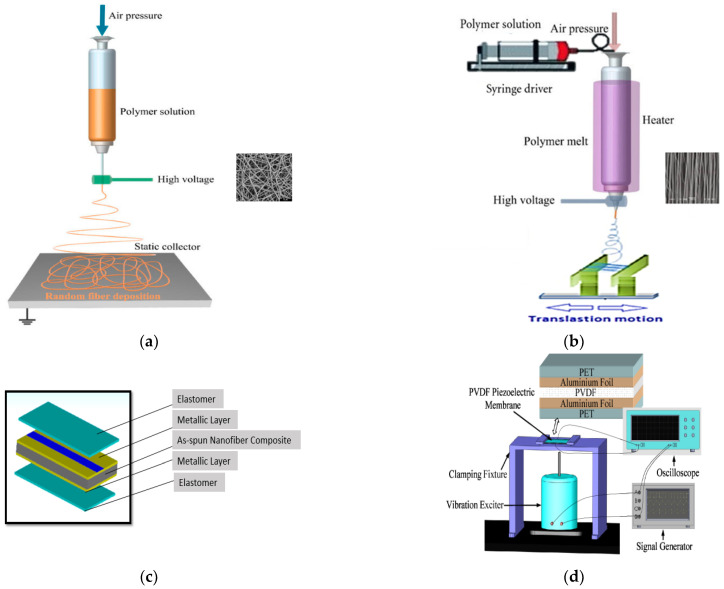
Schematic illustration of (**a**) conventional electrospinning configuration for nonwoven fibers, (**b**) modified electrospinning configuration with parallel electrodes and linear translation motion to produce aligned nanofiber mats, (**c**) the structure of the fabricated piezoelectric device with two elastomer sheets and two copper layers and one nanofiber composite membrane, and (**d**) piezoelectric measurement setup.

Electrospinning is one of the most capable and reliable methods for producing polymer nanofibers. Since the 1980s, it has been related to materials science and nanotechnology aspects and lately has drawn increasing attention [1]. The important feature of such a technique is its development for lots of applications owing to its easiness and truncated energy consumption [2,3,4,5]. Many modifications in the electrospinning experimental setup have been conducted, especially in the spinneret and the collector, to obtain a better morphology and characteristics of the spun fibers. The geometry of the collector drastically affects the features of the fibers. The ordinary collector is a metallic sheet to complete the electrical field generation and results in non-woven mats (see Figure 1a), while parallel collectors with a gap in between are the perfect collector to obtain aligned fibers [6]. For the electrospun fibers to be used in a wearable nanogenerator application, aligned nanofibers are preferred for a higher output power through the enhancement of piezoelectricity, mechanical properties, and electrical and thermal conductivities [7,8,9,10] owing to their high aspect ratio and large surface-area-to-volume ratio.

To handle the growing mandate for portable and flexible devices, piezoelectric energy harvesting technologies that generate electrical power from the applied mechanical force have been extensively explored for many functions such as control, sensing, micro-electromechanical systems, structure, military, and industrial [11,12,13,14,15,16,17,18,19].

Semiconductor piezoelectric materials such as CdS, ZnO, InN, GaN, and ZnS [20,21,22,23] have wide applications in converting mechanical energy from the environment, such as vibrations or body movements, into electrical energy [24]. Despite the brittle nature of this group as nano-generators and the difficulties of being integrated into fabrics, they can add self-powering features to the microelectronic devices. ZnO and ZnS are important wide bandgap semiconductors, and both have an extensive range of applications for optical and electric devices. Lately, many researchers have studied ZnO@ZnS nano-composites with various morphologies, such as nanorings, nanowires, nanoparticles, and other nanostructures [25,26,27,28,29,30].

On the other hand, Polyvinylidene fluoride (PVDF) has been ranked among piezoelectric polymers for its excellent piezoelectric qualities in addition to its cost-effective, biocompatibility, elasticity, and chemical stability [31,32,33,34] This polymer exhibits different polymorphic crystalline phases which are α, β, γ, and δ. Amongst these distinct polymorphs, α is plentiful, but the β phase donates PVDF, its piezoelectric feature, due to its huge spontaneous polarization and the virtue of its ferroelectric crystalline structure [35]. PVDF and copolymers [36] are better candidates as nanofibers for wearable energy devices than piezoelectric ceramics [37]. Nevertheless, its reduced piezoelectric coefficient has restricted their use in different applications. For an efficient nanogenerator, it is necessary to maximize the piezoelectric performance of the nanofibers. To do so, the electroactive crystalline β-phase must be increased [38]. However, the conventional fabrication methods of PVDF usually result in mats with a higher content of the α crystalline phase, which demands additional treatments of mechanical stretching and electric poling to transform the α to the β phase [39,40]. Many recent studies have reported the one-step production of PVDF piezoelectric nanofibers membranes as a harvesting energy device [41] since the extra treatments are unnecessary. During the electrospinning process, the strong applied electric field led to a good alignment of the molecular dipoles [42]. Therefore, electrospinning of PVDF nanofibers can transform the non-polar α phase to the polar β phase. Randomly oriented and aligned PVDF nanofibers are used in flexible devices for mechanical-to-electrical energy conversion. In nonwoven electrospun PVDF, the conversion rate was lowered owing to the arbitrary orientation of fibers, which contradict each other. The important feature of aligned nanofiber mats is the potential formed across the whole nanofiber membrane thickness [43,44]. Chang et al. verified the fabrication of PVDF nano-generators with an elevated conversion efficiency [45], while Pu et al. adopted the direct-write methodology to manufacture PVDF nanofibers to be used as actuators [46].

However, fibrous nano-generators still have a low efficiency; various researchers adopted another strategy to enhance the efficiency of PVDF nanofibers. Most of them used different types of nanoparticles, for instance, graphene, carbon nanotubes, and BaTiO_3_, but the most popular and valuable alternative to improve the performance of nanogenerators is ZnO [47,48,49,50]. The literature shows the usage of different types of ZnO nanoparticles in a PVDF matrix; for example, the addition of 2 wt % Co-doped ZnO NPs into (PVDF-HFP) nanofibers produces an elastic nanogenerator that achieves an output ∼45% higher than the plan polymer matrix [51], while only 7 wt % ZnO NPs into PVDF nanofibers drastically enhance the piezoelectric behavior [52]. Extra improvement of the β crystalline phase can be accomplished by ZnO nanorods [53]. Better nanogenerator performance of (PVDF-TrFE) nanofibers can be achieved by the addition of ZnO/GO mixtures [54,55].

This work fundamentally aimed to enhance the performance and efficiency of a wearable and durable nanogenerator by incorporating ZnO@ZnS core–shell nanoparticles into an aligned (PVDF) nanofibrous mat, using a modified electrospinning technique. The ZnO@ZnS nanoparticles were electrospun with the polymer solution to increase the piezoelectric coefficient. Moreover, the nanofiber mat was implemented by placing it between flexible spin-coated elastomer sheets to keep its durability and functionality. PVDF composite nanofibers were characterized through X-ray diffraction (XRD), scanning electron microscope (SEM), Energy-dispersive X-ray spectroscopy (EDS), and Fourier Transform Infrared (FTIR) spectroscopy. Melting temperature was measured by a differential scanning calorimeter (DSC), and a universal testing machine was used for tensile testing. The electrical resistance was measured by Keithley 2601a, while the piezoelectric behavior was measured using a lab-made experimental setup. To the best of our knowledge, this is the first report on fabricating wearable flexible membranes by adding ZnO-ZnS core–shell nanoparticles to a piezo-polymer. The ligh tweight, versatile membrane has a high potential to be used for nanogenerators.

**Significance of the work:** The production of highly aligned nanofibers via the modified electrospinning setup. To our knowledge, it is the first time that the linear repetitive motion of the parallel electrodes has been used while collecting the nanofibers. This cost-efficient and innovative idea drastically increased the alignment of the fibers, which could open up new horizons of applications that need highly aligned fibers such as tissue engineering. On the other side, incorporating the ceramic core-shell nanostructure enhanced the nanogenerator performance.

## 2. Materials and Methods

### 2.1. Materials

Poly (vinylidene fluoride) (PVDF) (Mw 275,000 g/mol), N, N-Dimethylformamide (DMF), and acetone (used as a solvent) were purchased from (Merck Chem. Co., Kenilworth, NJ, USA) and were used as received. The synthesized ZnO/ZnS core-shell NPs by (NanoTech, Cairo, Egypt) showed a roughly spherical shape with a mean diameter of 10 nm.

### 2.2. Preparation of the Nanofiber Mat by Modified Electrospinning

The polymer solution was prepared by dissolving PVDF into a solvent mixture of DMF/acetone at a concentration ratio of 6/4 (wt/wt) and gently stirred for 3 h at 55 °C. The obtained solution achieved a concentration of 26% (wt/wt) ZnO@ZnS core-shell nanoparticles at concentrations of 5, 8, and 10 wt %. Subsequently, the prepared solutions were stirred at 55 °C for 45 min to be ready for the electrospinning process. A lab-made electrospinning apparatus [56] was used to obtain the nanofibers. The electrospinning apparatus involved a syringe of volume (5 mL) equipped with a stainless-steel needle directly connected to the positive electrode. The flow rate was controlled by a syringe pump, while the ground pen of a DC power supply (15−30 kV) was connected to the modified collector.

The modified collector for the aligned fibrous membrane was fabricated in the form of two metallic sheets of dimensions (160 × 1.25) mm and separated by a small gap. The experimental setup is shown in Figure 1b. The experiment conditions were controlled through many experimental attempts to obtain aligned uniform fibers. For example, some pre-experiments were performed to electrospin the composite on the collector with different gap sizes to achieve the best results. Afterwards, fibers of PVDF, PVDF-with varying weight percentages of ZnO@ZnS nanoparticles, were made at three different voltages, 14, 18, and 22 kV, to pick the functional voltage. Finally, the best results were obtained at a 3 cm collector plate separation distance and an applied voltage of 18 kV. To avoid material loss and contain the fiber formation inside the collector, all electrical parts in the experiment area were isolated. The PVDF solution was packed into the syringe pump at room temperature. The spinning process took the vertical configuration concerning the collector at a distance of 12 cm and 0.4 mL/h volume flow rate. In light of the chosen nanogenerator application, it was particularly important to obtain higher values of the piezoelectric coefficient of the PVDF composite fibers. The piezoelectric response of a composite nanofiber hangs on the electric field between the needle and the collector, the feeding rate, and needle size. However, the thickness of the mats and the applied electric field predominantly control the piezoelectric performance. Therefore, all experimental conditions and parameters were controlled to keep the thickness of the mats almost the same with and without ZnO@ZnS CS NPs addition. The electrospinning time was adjusted to 7 min to study the relationship between the concentration of ZnO@ZnS nanoparticles and some important features such as the mat’s thickness, the average diameter of the nanofibers, and β phase content, as illustrated in Table 1.

### 2.3. Piezoelectric Device Fabrication

The PDMS elastomer sheet was cast on a flat surface of roughly 850 μm thickness by using the ratio of the resin to the curing agent 10:1. The curing process took almost 6 h at a temperature of 60 °C. After that, the sliced square PDMS sheets of area 2 × 2 cm^2^ experienced a Cu sputtering process through vapor deposition to prepare the PDMS-based electrodes with a copper layer of 220 nm in thickness. The job of the copper electrode was to act as good conductive contact between the copper wires and nanofibrous layer to assemble the nanogenerator. The conductive layer was evaporated into the elastomer substrate to improve the adhesion, which decreased the contact resistance with the device’s supportive membrane and the copper connectors. The PVDF nanofiber mat containing ZnO@ZnS nanoparticles (PVDF-5, 8 and 10 wt % ZnO@ZnS) was sandwiched between two Cu-sputtered PDMS elastomer sheets. At the same time, the copper connectors were pasted to the electrodes by conductive paste (Figure 1c). The compact device was finally pressed and sealed with commercial paper tape. A high voltage was applied for almost 2 h across the terminals of the copper connectors to pole the nanofibers. The versatile wearable energy conversion device was achieved thanks to the elastomer PDMS layers that give the nanogenerator its variability and flexibility. In addition, the very thin design was proposed to guarantee the device’s compliance with the idea of being attached to different types of clothes or textiles.

### 2.4. Materials Characterization

The synthesized ZnO@ZnS nanoparticles were characterized by transmission electron microscope (TEM; FEI Tecnai G20 operated at 200 kV), while the as-spun fibers were observed using scanning electron microscopy (SEM; SU8010, Hitachi, Kyoto, Japan) after gold sputtering. To complete the morphology characterization of the fabricated mats, Energy-dispersive X-ray spectroscopy (EDS) with elemental mapping was conducted. IR spectroscopy was carried out with an FTIR spectrophotometer (Nicolet 6700, Thermo Electron Co., Waltham, MA, USA). The specific surface area of the core–shell nanoparticles was determined by the Brunauer−Emmett−Teller (BET) method. X-ray diffraction (Expert Philips diffractometer with Cu Kα radiation) was utilized to explore the crystalline phases of the composite. A differential scanning calorimeter (DSC) (model: DSC 2010, TA Instruments. Co., New Castle, DE, USA) was used to measure the melting enthalpy (ΔHm) of PVDF and nanofibers with different concentrations with a heating rate of 20 °C/min. DSC experiments were performed in a nitrogen atmosphere as it has a higher heat conductivity and thus heats in the chamber of the sample in an evenly distributed way.

Tensile tests were performed at room temperature using a Zwick tensile testing machine (Model Z010, Ulm, Germany). Samples were prepared in dimensions of 80 × 10 × 2 mm^3^. A hydraulic press was utilized to prepare the samples under a pressure of 150 kg cm^−2^. A constant crosshead speed of 10 mm/min was selected according to ISO, 37 and the stress–strain data were recorded up to the failure of samples. At least five measurements from each sample were recorded, and the average values were reported.

### 2.5. Device Characterization

Keithley (keithley 2601a) with an input voltage between −42.5 V and +42.5 V was used to measure the electrical resistance of the fibers while the impedance was measured by an impedance analyzer. The experimental setup shown in Figure 1d illustrates the measurement of the piezoelectric output voltage of the wearable device samples. The function generator was connected to a vibration exciter while the electrically insulated sample was linked to a digital oscilloscope to measure the voltage. For comparison, a very thin layer of the PVDF was fabricated, poled, and measured using the same setup. For the practical application of the device, the open circuit voltage as a function of time in seconds was measured as a response to the mechanical force of 4 N as the input stress to simulate the mechanical stresses of compressions and release.

## 3. Results and Discussion

### 3.1. Characterization of the ZnO@ZnS Core–Shell Nanoparticles

**Figure 2:**
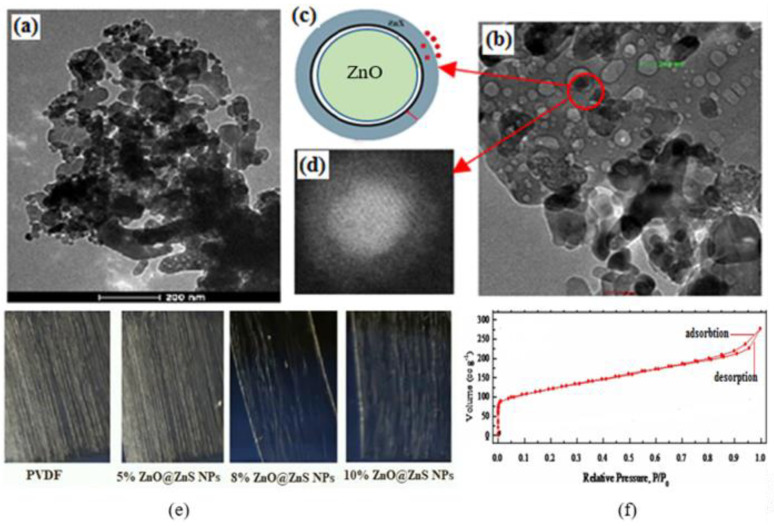
Structure of ZnO@ZnS CS NPs, (**a**,**b**) bright-field TEM images with different magnifications of the particles, (**c**) diagrammatic representation of the core–shell nanostructures, (**d**) high-resolution TEM image of ZnO core and ZnS shell, (**e**) photographs of the aligned spun fibers of pure PVDF and its composites with concentrations (5%, 8%, 10%) of ZnO@ZnS CS NPs (all photos with a scale bar of 150 μm), and (**f**) the nitrogen adsorption–desorption isotherm of the ZnO@ZnS CS NPs.

The schematic and TEM images in Figure 2 illustrate the characteristics of ZnO@ZnS CS NPs particles. It became clear that two different materials were present in the outer and inner layer by observing the apparent contrast between the color of the shell and the core, where the core was darker in grey color than the shell part, which implied the presence of ZnO in core and ZnS as the shell (see HRTEM image Figure 2d).

The TEM image in Figure 2a,b shows the grain of ZnO@ZnS CS NPs. The HRTEM shown in Figure 2d denotes that the dimensions of the ZnO core were approximately 80 nm in diameter, while the ZnS shell layer had a thickness of about 29 nm. The nitrogen adsorption–desorption isotherm shown in Figure 2f exhibited a slightly narrow hysteresis at higher pressures indicating the existence of silt pores. The specific surface area was about 1106 m^2^/g.

### 3.2. Morphology of the Fabricated Composite Nanofibers

Since stable and appropriate piezoelectric content is related to the presence of uniform thin nanofiber mats, it is important to optimize the electrospinning process to achieve thinner mats and keep reasonable β-phase crystallinity. Despite the conventional chaotic motion of the electrospinning jet, the parallel plate collector in our setup configuration (moving on the top of a liner stage to accomplish frequent forward and backward motion) provided excellent aligned fibers by strengthening the stretching ratios of the polymer jet. Figure 2e shows the optical microscope photos of as-spun PVDF nanofibers and the composite of PVDF with a different weight percentage of ZnO@ZnS CS NPs. As shown in the figure, the spinning process was much easier with a low percentage of nanoparticles, and the mat seemed denser with a thinner fiber diameter. In contrast, the increase in the nanoparticles’ weight percentage increased the diameter of the fibers and decreased the fabricated mat densities.

**Figure 3:**
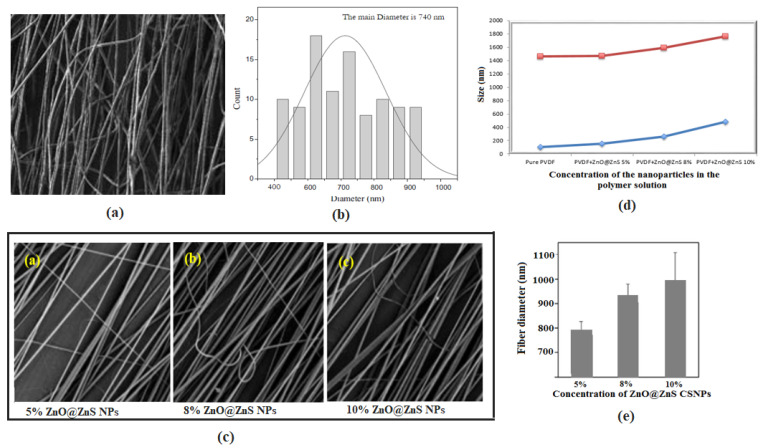
SEM micrograph of aligned nanofibrous mats, (**a**) pure PVDF, (**b**) diameter distribution of the pure PVDF (150 points from the SEM image were used to calculate the average diameters), (**c**) SEM micrographs of aligned nanofibers of composite PVDF+ (a) 5 wt %,(b) 8 wt %, and (c) 10 wt % of ZNO@ZnS CS NPs (all SEM images with a scale bar of 20 μm), (**d**) comparison between the size of the fibers with minimum fiber diameters (blue curve) and maximum fibers diameters (red curve) with a different concentration of nanoparticles, (**e**) the variation of the average diameters of the fibers versus the concentration of the ZNO@ZnS CS NPs.

SEM images of PVDF and PVDF-ZnO@ZnS CS NPs fibers are illustrated in Figure 3. A high alignment and uniform fibers can be observed from the SEM images, and the size distribution study gave a range of diameters of the PVDF sample from 500 to 1000 nm with a mean size of 740 nm. The addition of ZnO@ZnS CS NPs in the PVDF matrix caused a slight change in the mean diameter of the fibers, as shown in the bar chart of Figure 3e as the mean diameter increased to 790, 925, and 1050 nm with the solution concentrations of 5, 8, and 10 wt %, respectively (Table 1). It was also noticed that the moving parallel plate collector provided aligned fibers with no arrangements of beads for a long range of surface area in all fabricated mats, which increased the efficiency of the suggested modification of the spinning technique.

It was observed in the previous studies that the addition of nanoparticles could change the optimized conditions of the experiments. In these images, the number of deposited fibers decreased with the increase in the nanoparticles and this may have been due to the electrostatic repulsion force that exists with passing time [57]. On the other hand, the fiber diameters increased when the concentration of ZnO@ZnS CS NPs increased in the PVDF solution (see Figure 3e) and this may have been due to the unstable density of charges through the Taylor cone. This was furthermore confirmed by the graphs in Figure 3d, which indicated a comparison between the size of the fibers with minimum fiber diameters (blue curve) and maximum fiber diameters (red curve) with different concentrations of nanoparticles. The minimum and maximum diameters corresponding to each nanoparticle’s concentration were obtained from histograms of each concentration.

### 3.3. FTIR and EDS Analysis of the Composite Nanofibers

**Figure 4:**
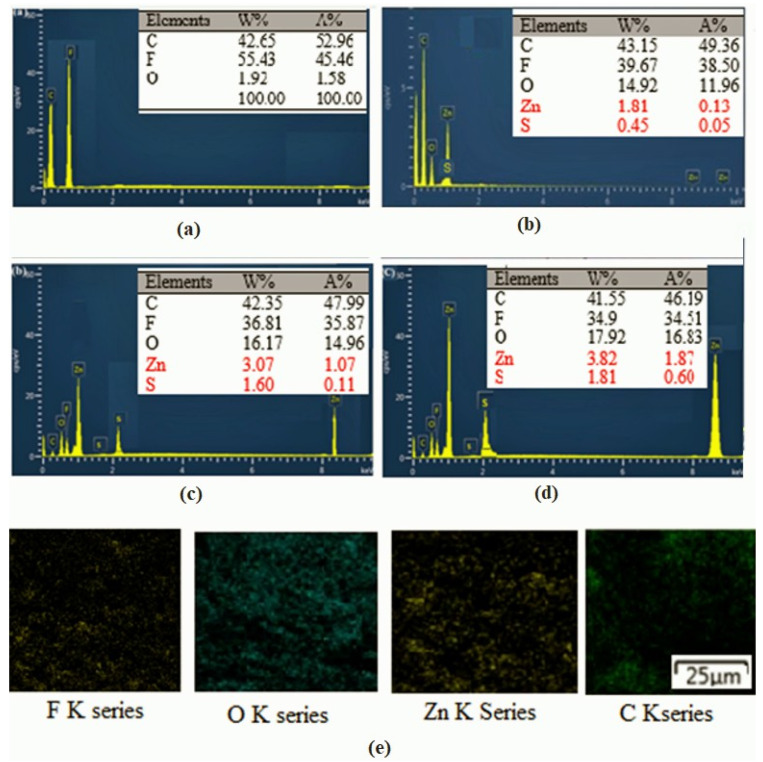
EDS scanning spectra of PVDF-ZnO@ZnS CS NPs composite mats as a function of varying NPs content: (**a**) 0 wt %, (**b**) 5 wt %, (**c**) 8 wt %, and (**d**) 10 wt %. (**e**) EDX elemental mapping of PVDF-10 wt %.

Figure 4 shows the EDX spectrographs of PVDF and composite nanofibers which support the presence of elements of sulfur, oxygen, and a rich atomic ratio of zinc. The additive core–shell ZnO@ZnS nanoparticles are hydrophilic [58], but PVDF is a hydrophobic polymer [59]. For this reason, during the spinning process and drying of the membrane may, some of the nanoparticles appeared at the outer surface of the fibers and were obviously mapped in the EDS results.

It is well known that PVDF presents in various crystalline phases; among them, β and α phases are the most popular. A distinctive absorption in the IR region is a characteristic property of the crystalline phases of PVDF. Therefore, IR spectroscopy can be used to characterize them in the range from 1500 to 400 cm^−1^.

**Figure 5:**
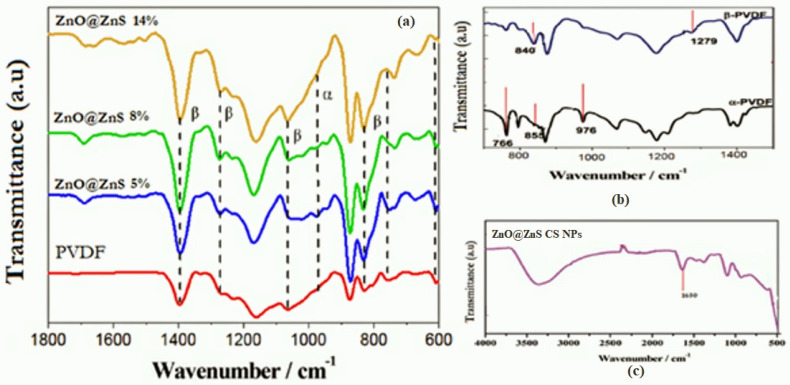
Analysis of the FTIR spectra, (**a**) PVDF-ZnO@ZnS CS NPs composite mats as a function of varying NPs content from 0 wt % to 10 wt %, (**b**) FTIR spectra of PVDF nanofibrous mats (α and β phases distinctive transmission bands), and (**c**) FTIR spectrum of ZnO@ZnS CS NPs.

The characteristic bands shown in Figure 5b are the IR spectra of the PVDF nanofibers sample, and they were observed at, 764, 853, and 975 cm^−1^ for the α phase, while for β phase bands at 840, 1279 cm^−1^ were found [60,61]. The contribution of ZnO@ZnS nanoparticles in PVDF phase transition can clearly be seen from the weakening of α phase band intensities versus the enhancement of the absorption band intensities of the β phase, as illustrated in Figure 5a.

The absorption peak at wavelengths 840 cm^−1^ and 764 cm^−1^ of the α and β phases, respectively, were estimated to determine the beta crystalline phase % according to the following equation [62,63].

$$\beta -\mathrm{phase}\;\left(\%\right)=\frac{A_{\beta }}{1.26A_{\alpha }+A_{\beta }}\times 100$$

where *Aα* and *Aβ* represent the intensities of the *α* and *β* phases at 840 and 764 cm^−1^, respectively. The impact of ZnO@ZnS CS NPs on the content *β*-phase is mentioned in Table 1.

Stimulation of the edge-on ordering of the crystal by the interaction of the hydrogen bonds may have been the reason behind the growth of the *β* phase. The *β* phase remarkably increased by 17% due to the addition of ZnO@ZnS nanoparticles, while other studied additives achieved a 9% increase by ZnO nanoparticles [64].

### 3.4. Crystalline Structure of the Composite Nanofibers

**Figure 6:**
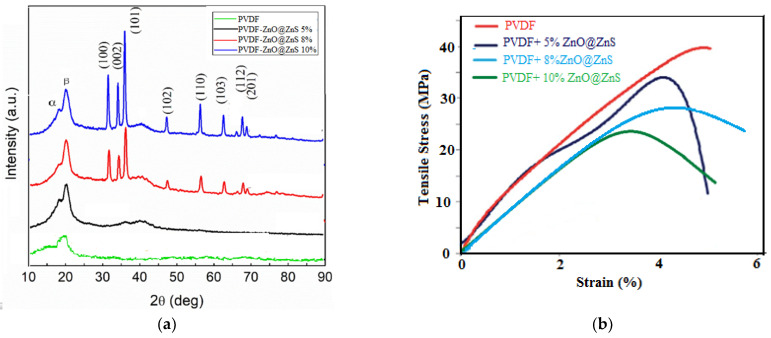
Characterization of pure PVDF and PVDF-ZnO@ZnS CS NPs composite nanofibrous mats as a function of varying NPs content from 0 wt % to 10 wt %. (**a**) XRD patterns, (**b**) Engineering stress–strain curves.

The crystallinity of the fibrous mats was studied by X-Ray diffraction spectra. The crystalline patterns of PVDF and PVDF-ZnO@ZnS CS NPs composite nanofibers are shown in Figure 6a. The peaks corresponding to angles 33, 34, 35, 45, and 45° elucidated the existence of ZnO@ZnS CS NPs in the polymer composite and confirmed the predominant existence of the beta phase. The XRD peak appeared at an angle of 18.8° corresponding to the α phase, while the main peak observed at an angle of 20.5° was attributed to the existence of the β phase. The intensity of the β phase peak increased with the increase in the concentration of ZnO@ZnS nanoparticles, indicating that incorporation of the ZnO@ZnS NPs excellently enhanced the pattern of the β crystalline phase and enriched the piezoelectric behavior of the membranes. In addition, the high alignment configuration through electrospinning and stretching of the fibers in the presence of the high voltage offered a good opportunity to transform the α-phase into the β-phase [65,66].

### 3.5. Mechanical and Thermal Properties of the Composite Nanofibers

Figure 6b shows the representative stress–strain curves of PVDF and its composite nanofibrous mats, PVDF + (5%, 8%, 10% ZnO@ZnS CS NPs). The thermoplastic behavior was predominant in all samples with reasonable ductility. However, all mechanical properties of the pure polymer were slightly altered by the addition of nanoparticles. Young’s modulus, elongation at break, and fracture stresses are all summarized in Table 1. It can be seen that Young’s modulus was reduced by 13.8% while the highest reduction in elongation at rupture was at 28% with the maximum ZnO@ZnS nanoparticles content. It was found that the lower Young’s modulus values of the polymer composite, the higher the efficiency of the conversion of mechanical into electrical energy, hence the best performance (higher generated voltage) of the nanogenerator. The reduction in the composite flexibility may be attributed to the brittle nature of the additive nanoparticles, which limited the moving of the chain segments of the PVDF polymer. From a macroscopic point of view, the nanoparticles acted as defects and prevented the PVDF chains from packing with each other. As a result, the tensile strength of the polymer matrix became less pronounced and, at the same time, decreased the elongation at break in the composites than those of the pure polymer matrix [67,68].

**Figure 7:**
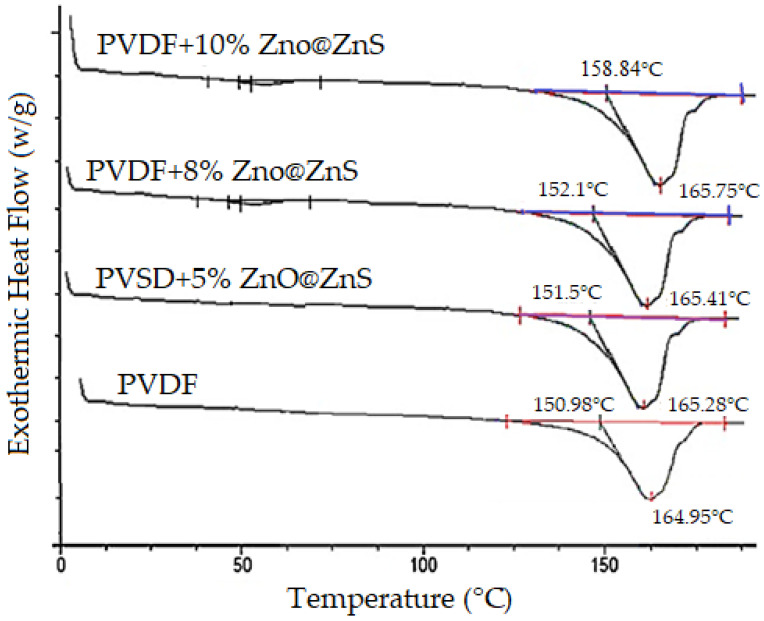
DSC thermograms of electrospun pure PVDF and PVDF-ZnO@ZnS CS NPs composite nanofibrous mats as a function of varying NPs content from 0 wt % to 10 wt %. (with a heating rate of 20 °C/min).

DSC melting thermograms of the PVDF and PVDF-ZnO@ZnS CS nanofibers mats are shown in Figure 7. It can be seen that the different concentrations of nanoparticles in the polymer composite showed shifted melting endotherms. As reported, this may have been due to the polymorphism, and/or variation in morphology. However, the dominant idea is associated with the change of the polymorphic structure of PVDF with the increase in the nanoparticles [69,70]. It can be noticed that the enthalpies of PVDF-ZnO@ZnS SC composite nanofibers were (ΔHm = 54.21, 55.13, and 56.94, J/g) corresponding to the nanoparticle concentrations of 5, 8, and 10%, respectively, which were more than that of the pure PVDF nanofiber mat (52.86 J/g).

The degree of mass crystallinity, $X_{c}$ (%), can be calculated according to the following equation [71]:

$$X_{c}=\frac{\Delta H_{m\left(sample\right)}}{\Delta H_{Lit}}\times 100$$

where $\left(\Delta H_{Lit}=103.4\;J/g\right)$, Δ*H_Lit_* is the enthalpy of melting for a 100% crystalline material. As a result, the significant increase in crystallinity for composite nanofiber mats was 56.8%, while for PVDF nanofibers, it was 51.4% which confirmed the formation of a new crystalline structure (β phase) in the electrospun nanofiber mats.

### 3.6. Impedance and Electrical Conductivity of the Nanogenerator Device

The significant spread of the small number of nanoparticles into the polymer nanofibers matrix can form a strong conductive network with intrinsic conductivity. Consequently, this facilitates electron migration and provides more charge transfer channels. This behavior remarkably reduces the internal resistance of the composite nanofibers, which reduces the polarization electric field and provokes the flow of electrons. Thus, the overall electric output of the composite nanofibers is readily enhanced. For the mentioned reasons, it is important to reduce the resistance in a wearable nanogenerator. The electrical resistance of the device fabricated from PVDF and PVDF-ZnO@ZnS CS NPs nanofibers was measured by a Keithley instrument to analyze and assess the performance of the fabricated device and to study the effect of the nanoparticles added to the polymer matrix. As can be seen in Table 2, a remarkable reduction in fiber impedance was achieved by increasing the ZnO@ZnS NPs concentration. It was observed that the order of electrical resistance of electrospun PVDF nanofiber mats was around 300 MΩ. In comparison, adding 10% of nanoparticles in the composite nanofibers reduced the resistance to 10 MΩ. The semiconductor nature of the ZnO@ZnS nanoparticles may have been the reason behind this resistance reduction.

**Figure 8:**
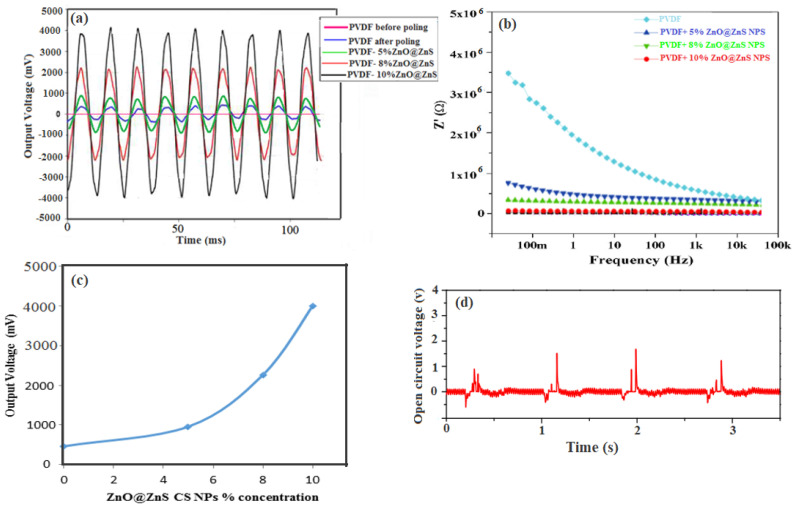
Electric properties of the nanogenerator device, (**a**) instantaneous waveform of the piezoelectric response of PVDF thin film (before and after poling) and PVDF-ZnO@ZnS CS NPs composite nanofibrous mats as a function of varying NPs content from 0 wt % to 10 wt %, (**b**) the experimental amplitude impedance of the piezoelectric electrospun PVDF and composite nanofiber device as a function of frequency at room temperature, (**c**) output voltage of the electrospun nanogenerator as a function of nanoparticles concentrations, (**d**) open circuit voltage as a function of time recorded in the range of seconds.

Figure 8b shows the amplitude impedance of the composite nanofibers which was measured via an impedance analyzer with a frequency range of 1–10^6^ Hz at room temperature under an applied voltage of 0.3 V. In general, the impedance was reduced by increasing the applied frequencies, which indicated good capacitance behavior of the nanogenerator device. A significant reduction in the amplitude impedance was also observed with an increase in the NPs concentration in the composite nanofiber; such behavior indicated that the large specific surface area of NPs was more valuable in increasing the interface area, which enhanced the conductivity.

### 3.7. The Piezoelectric Response of the Nanogenerator Device

The piezoelectric response of electrospun composite PVDF fibrous mats revealed the ultimate efficiency of the mechanical-to-electrical energy conversion of the nanogenerator. The sensitivity of the nanogenerator can be described as the ratio of the charge generated by the PVDF mat to the normal excitation force. In other words, this may simulate the piezoelectric coefficient, which is related to the electric field produced by mechanical stress. The output voltage of PVDF thin film (before and after poling) and the composite nanofibers with different concentrations of ZnO@ZnS CS NPs were measured under excitation voltage 1 V and vibrational frequency 7 Hz to investigate the electrical response of the wearable nanogenerator. This range frequency was chosen to mimic human activities and to verify the wearable applications. The measurements were repeated for each face of the device three times, and the mean value of six trials was used to ensure the reliability of these results. The output voltage was measured from a PDMS elastomer sheet with two copper electrodes with the same experimental conditions to assure that voltage output only resulted from the nanofibers mats. The result declared no signal voltage output from PDMS. Results of the output voltage of the tested samples are shown in Figure 8, where the samples showed a remarkable piezoelectric response.

Figure 8a illustrates the output voltage of PVDF thin film and the PVDF composite nanofibers mats recorded by the oscilloscope versus time in the millisecond scale. The PVDF thin film without any electric poling did not respond (see Figure 8a). The poled nanofiber of PVDF recorded a maximum output voltage of 532 mV. At the same time, the incorporation of ZnO@ZnS CS NPs content by 5%, 8%, and 10% increased the electrical response to 1.02 V, 2.33 V, and 4.42 V, respectively, as shown in Figure 8c. The conversion sensitivity of the PVDF fabricated device recorded almost 0.091 V/N·mm^3^, while that of the PVDF–10 wt % ZnO@ZnS composite mat recorded a sensitivity of 0.153 V/N·mm^3^ which is better that the best energy harvester devices reported in the previous work [72]. A comparison of the presented piezoelectric device performance with previously reported data is presented in Table 3. This highly improved performance was mainly due to the superior piezoelectric phase provided by various factors; the intrinsic piezoelectric properties of the core–shell structure of the ZnO@ZnS nanoparticles [73], the electromechanical drawing during the electrospinning process, and the aligned nanofibers mats thanks to our modified spinning configuration led to a high degree of transformation from the α-phase to the β-phase.

## 4. Implementation of the Nanogenerator Device

To test the reliability and sensitivity of the PVDF-ZnO@ZnS NPs composite nanogenerator device, compression increase and release under applied pressure tests were performed continuously. Figure 8d shows the pressure increase and release test of PVDF-10 wt % ZnO@ZnS NPs nano-generator. The developed large positive peak of open circuit voltage under applied stress may be attributed to the alignment of the molecular dipoles through the spinning process. At the same time, the damping effect may explain the presence of the –ve peak [75]. The change in strain of the nanofiber mat concerning time was the reason for the uneven response between the press and release of the input stress. The curve also shows that the device exhibited a favorable recovery time when the pressure was released due to the high elasticity of the PVDF matrix. It is also noted that the stability of the device was likely improved as the output voltage of the first cycle was much lower than the following cycles [74].

## 5. Conclusions

High-performance piezoelectric composite Polyvinylidene fluoride/ZnO@ZnS core–shell nanoparticle membranes were produced by a modified electrospinning setup. The modified collector technique enhanced the stretching of the nanofibers to be aligned in good shape and, at the same time, supported in situ poling, which resulted in a crystal transformation from the α-phase to the β-phase. A PVDF solution of 26% (wt/wt) was prepared under standard conditions, and ZnO@ZnS CS NPs with weights of 5, 8, and 10% wt were added. SEM, FTIR, EDS, XRD, and DSC were used to characterize the produced nanofibers mats, while PVDF mats were evaluated as wearable nanogenerators, thereby testing the respective piezoelectric performance. The morphological SEM characterization images confirmed the ability of the modified spinning technique to produce well-aligned nanofibers mats. XRD, FTIR, and DSC revealed an improvement in the crystal structure of the nanofibers related to the addition of the core–shell nanoparticles. The piezoelectric response based on applied mechanical vibrations on the fabricated device showed that the aligned composite nanofibrous mats significantly improved the electrical output signals. Finally, the results from assessing the fabricated composite nanogenerator device demonstrated the flexible design of power sources as a wearable textile without additional treatments. The modified collector of the spinning setup provided an acceptable control of the crystalline phases of nanofibers. The smart design of the device using elastomer sheets and connecting electrodes to the fibrous mats improved the flexibility and workability of such devices. The mechanical to electrical efficiency of the generator device was improved significantly by adding the nanoparticles.

## Figures and Tables

**Table 1:** The effect of ZnO@ZnS core–shell NPs concentration on fiber diameters, content of β-phase with almost constant membrane thickness of 14 μm, and the mechanical properties of the nanofibrous mats.

ZnO@ZnS.CS NPs (wt %)	Fiber Diameter (nm)	β-Phase Content (%)	Mechanical Characteristics		
			Young’s Modulus (MPa)	Fracture Stress (MPa)	Elongation at Break (%)
0	740	68	1640 ± 0.63	46 ± 0.59	6.1
5	790	75	1595 ± 0.37	41± 0.57	5.5
8	925	79	1507 ± 0.88	34± 0.49	4.9
10	1050	81	1413± 0.64	29± 0.31	4.6

**Table 2:** The electrical resistance of the PVDF and PVDF-ZnO@ZnS CS NPs composite nanofibrous mats as a function of varying NPs content from 0 wt % to 10 wt %.

ZnO@ZnS Nps %	F(x) = P_1_ × X + P_2_		R^2^	Resistance Ω
	P_1_	P_2_		
0	2.425 × 10^8^ (2.416 × 10^8^, 2.476 × 10^8^)	−24.69 (−23.47, −25.38)	0.863	3.35 × 10^8^
5	1.047 × 10^8^ (1.025 × 10^8^, 1.094 × 10^8^)	−14.43 (−14.95, −13.424)	0.975	0.15 × 10^8^
8	4.196 × 10^7^ (4.1263 × 10^7^, 4.21 × 10^7^)	−7.042 (−7.581, −6.649)	0.969	5.9 × 10^7^
10	5.028 × 10^6^ (5.067 × 10^6^, 5.311 × 10^6^)	−5.004 (−5.621, −4.822)	0.991	0.1 × 10^7^

**Table 3:** Comparison of the piezoelectric device performance with previously reported data.

Piezoelectric materials	Max wt %	With/without Poling	F(β)%	Max Output Voltage (V)	Ref.
PVDF	-	without	7%	0.028	[6]
ZnO NWs/PVDF	-	with	4%	0.2	[4]
PVDF/Carbon nanofiber	0.5% (w/w)	with	11%	0.56	[19]
PvDF + MWCNT	5 wt %	without	-	6	[15]
titanium dioxide- rGO/PVDF	2.5 wt %	with	10%	3.9	[16]
	2.5 wt %				
Li doped ZnO NW-Polymer Composite	3 wt %	with	9%	180	[2]
Fe-fGO/PVDF	5 wt %	without	14%	1.2	[10]
Native Cellulose microfiber	8 wt %	without	-	30	[60]
BaTiO_3_ nanotubes		with	-	3.5	[74]
ZnO@ZnS CS NPs/PVDF	10 wt %	with	17%	4.42 sensitivity of 0.153 V/N·mm^3^	Present work

## Data Availability

The raw/processed data required to reproduce these findings cannot be shared as the data also form part of an ongoing study.
